# Prevalence of Fasciolosis and Dicrocoeliosis in Slaughtered Cattle and Sheep in Siirt Province, Türkiye: A Comparison of Coprological and Postmortem Findings

**DOI:** 10.3390/vetsci13080738

**Published:** 2026-07-25

**Authors:** Muhammed Yasul, Milad Afşar, Ali Bilgin Yilmaz, Muhammed Ahmed Selcuk, Mahsa Torkamanian-Afshar, Hasan Yilmaz

**Affiliations:** 1 Home Health Care Program, Health Care Services Department, Bartın University School of Health Services, Bartın 74100, Turkey; 2 Division of Medical Parasitology, Department of Basic Medical Sciences, Faculty of Medicine, Van Yüzüncü Yıl University, Tuşba, Van 65080, Turkey; mtmilad@gmail.com (M.A.); hasanyilmaz@yyu.edu.tr (H.Y.); 3 Faculty of Health Sciences, Van Yüzüncü Yıl University, Tuşba, Van 65080, Turkey; alibilginyilmaz@yyu.edu.tr; 4 Department of Parasitology, Faculty of Veterinary Medicine, Siirt University, Siirt 56100, Turkey; ahmed.selcuk@siirt.edu.tr; 5 Software Engineering, Faculty of Engineering and Natural Sciences, İstanbul Topkapı University, Istanbul 34020, Turkey; mahsatorkamanianafshar@topkapi.edu.tr

**Keywords:** cattle, *Dicrocoelium dendriticum*, *Fasciola* spp., postmortem examination, prevalence, sedimentation, sheep, Siirt

## Abstract

This study aimed to determine the prevalence of the liver trematodes *Fasciola* spp. and *Dicrocoelium dendriticum* in cattle and sheep slaughtered in Siirt Province using fecal sedimentation and postmortem liver examination to compare their detection performance, and to estimate the sensitivity of fecal sedimentation relative to postmortem examination. A total of 2211 animals, comprising 374 cattle and 1837 sheep, slaughtered during the study period, were included in the study. Fecal samples were collected rectally before slaughter, and after slaughter, the liver, gallbladder, and bile ducts were examined macroscopically. Fecal samples were analyzed using sedimentation; postmortem examination was the reference method. Based on postmortem examination, *Fasciola* spp. and *D. dendriticum* were detected in 26.2% (98/374) and 7.2% (27/374) of cattle, and in 14.8% (271/1837) and 5.9% (108/1837) of sheep, respectively. Fecal examination detected significantly fewer infections for both parasites in both hosts (McNemar test, *p* < 0.001 for all comparisons). The sensitivity of fecal sedimentation was 80.6% for *Fasciola* spp. and 29.6% for *D. dendriticum* in cattle, and 86.7% and 73.1%, respectively, in sheep. The prevalence of *Fasciola* spp. was significantly higher in cattle than in sheep (OR = 2.05, 95% CI: 1.58–2.67; *p* < 0.001), whereas the difference in *D. dendriticum* prevalence between cattle and sheep was not statistically significant (OR = 1.24, 95% CI: 0.80–1.92; *p* = 0.274). In cattle, age older than two years was significantly associated with *Fasciola* spp. (adjusted OR = 15.82, 95% CI: 8.12–30.84; *p* < 0.001) and *D. dendriticum* infection (adjusted OR = 3.29, 95% CI: 1.36–7.96; *p* = 0.008). In sheep, the Morkaraman breed had lower odds of *Fasciola* spp. infection (adjusted OR = 0.42, 95% CI: 0.32–0.56; *p* < 0.001) and *D. dendriticum* infection (adjusted OR = 0.23, 95% CI: 0.14–0.38; *p* < 0.001) than the Akkaraman breed, while age was significantly associated only with *D. dendriticum* infection (adjusted OR = 1.84, 95% CI: 1.25–2.71; *p* = 0.002). These findings demonstrate that liver trematode infections were common among slaughtered animals in Siirt. Reliance on fecal sedimentation may considerably underestimate prevalence, particularly for *D. dendriticum* in cattle. Age- and breed-related associations should be considered in surveillance and control programs. From a public health perspective, awareness of zoonotic fasciolosis and risks associated with consuming raw aquatic plants from potentially contaminated areas should be increased.

## Simple Summary

Liver flukes are parasitic worms that infect the livers of cattle and sheep, causing health problems in animals and reducing meat and milk production. Some of these parasites can also infect humans. This study examined the prevalence of these infections in cattle and sheep in the Siirt region of Türkiye. The researchers examined feces and livers from slaughtered animals to detect the parasites. They found that more than 33% of cattle and 20% of sheep had liver fluke infections. Cattle were more likely to be infected than sheep, and older cattle had higher infection values than younger ones. Among sheep, one breed (Akkaraman) was more infected than another (Morkaraman). The study also revealed that coprological examination alone frequently underestimates infection values, particularly for *Dicrocoelium dendriticum*. This limitation is mainly attributed to the small size of *D. dendriticum* eggs, which makes them highly prone to loss during routine sedimentation procedures. Because *Fasciola* spp. can also infect humans, local residents should avoid consuming raw aquatic plants such as watercress. These findings can help veterinarians and public health officials design more effective strategies to control liver fluke infections in the region.

## 1. Introduction

Fasciolosis is recognized by the World Health Organization (WHO) as an important foodborne trematode infection and continues to pose a public health risk. Dicrocoeliosis is also a zoonotic liver trematode infection, although human infections are reported relatively rarely. Fasciolosis is caused by species of the family Fasciolidae, namely *Fasciola hepatica* and *F. gigantica*. These liver trematodes cause substantial productivity losses in animals, particularly in endemic regions, and constitute an infection risk for humans. Dicrocoeliosis, on the other hand, is caused by *Dicrocoelium dendriticum*, which parasitizes the liver. It has been reported that *D. dendriticum is* encountered at lower frequencies in humans and exhibits lower pathogenicity than *Fasciola* species [1,2,3]. Adult *D. dendriticum* resides in the small bile ducts and does not migrate through the liver parenchyma. However, heavy infections can lead to bile duct hyperplasia, cholangitis, and cirrhosis, resulting in reduced weight gain and milk production [4].

Domestic and wild ruminants, particularly sheep, goats, cattle, and buffalo, are the principal definitive hosts of *Fasciola* spp.; humans and several other mammals may also be infected occasionally. Freshwater snails belonging to the family *Lymnaeidae* serve as intermediate hosts, although the relevant snail genera and species vary according to the *Fasciola* species and geographical region. Within the snail, the parasite undergoes larval development, after which cercariae are released and encyst as metacercariae on aquatic vegetation or other substrates. In contrast, *D. dendriticum* requires two intermediate hosts—terrestrial snails as the first intermediate hosts and formicine ants, particularly *Formica* spp., as the second intermediate hosts—although other ant genera may also be involved. Transmission occurs when grazing animals accidentally ingest ants containing metacercariae. These differences in life cycles have important implications for the ecology and control of these parasites. The pathogenesis of fasciolosis depends on several factors, including the parasite species, parasite burden, isolate characteristics, and host species. Although infections caused by *F. hepatica* and *F. gigantica* may produce similar clinical manifestations, the severity of disease and the risk of biliary obstruction are influenced by the parasite burden, stage of infection, and host response [5,6].

During the migration of immature *Fasciola* spp. through the liver parenchyma, various pathological changes may occur, including inflammation, edema, hemorrhage, necrosis, and mechanical tissue damage. Once the flukes reach the bile ducts and mature, they may cause cholangitis, fibrosis, bile duct hyperplasia, and partial or complete biliary obstruction. These changes may disrupt bile flow and lead to associated clinical signs. In infected animals, fasciolosis causes economic losses, including reduced pregnancy values, decreased meat and milk production, irregular reproductive cycles, and low birth weight in offspring [4,7,8].

The diagnosis of liver trematodes infections can be established using different methods depending on the stage of infection, parasite load, and the purpose of the investigation. Diagnostic approaches include postmortem macroscopic examination, microscopic/coprological examination, serological assays, antigen-detection tests, and molecular methods. Because eggs are generally not shed in feces until the infection becomes patent, conventional coprological egg-detection methods have limited diagnostic value during the early stages of infection. Earlier detection may therefore be achieved using serological, antigen-detection, or molecular methods [9,10,11].

Siirt Province is located in southeastern Türkiye, where livestock production, particularly sheep and cattle farming, represents an important source of income for rural communities. The climatic and ecological conditions in certain parts of the province may favor the development and persistence of the intermediate hosts of liver trematodes. Therefore, this study aimed to determine the prevalence of liver trematode infections in cattle and sheep slaughtered in Siirt Province using fecal sedimentation and postmortem liver examination and to evaluate the implications of the findings for animal and public health.

## 2. Materials and Methods

The Van Yüzüncü Yıl University Local Ethics Committee for Animal Experiments issued an official exemption statement confirming that ethical approval was not required for this study, in accordance with its decision dated 30 April 2026 (Decision No. 2026/04-10). Following the issuance of the exemption decision, the study was initiated within a short period by collecting samples from animals slaughtered in Siirt Province. All cattle and sheep slaughtered on the sampling days were systematically included in the study without applying any exclusion criteria. This sampling approach resulted in a total of 374 cattle and 1837 sheep being examined. Sex was recorded for all animals, and the sample consisted predominantly of females (cattle: 346/374, 92.5%; sheep: 1729/1837, 94.1%). The age of each animal was determined using dental examination and farm records, and information on age, breed, and sex was recorded. Before slaughter, approximately 30–50 g of fecal material was collected directly from the rectum of each animal and placed in appropriately sized sterile screw-capped containers. The samples were transported under refrigerated conditions at 4 °C to the Research Laboratory of the Department of Parasitology, Faculty of Medicine, Van Yüzüncü Yıl University, within 10 h of collection. Following slaughter, the liver, gallbladder, and bile ducts of each animal were macroscopically examined for the presence of adult *Fasciola* spp. and *Dicrocoelium dendriticum*.

### 2.1. Microscopic Examination of Stool Samples

The classical sedimentation method, a routinely used copromicroscopic technique, was employed to detect liver trematode eggs in fecal samples collected from sheep and cattle [12]. Postmortem examination was considered the reference method for determining infection status. Briefly, 5–10 g of feces was homogenized in physiological saline, filtered through a 250 µm mesh, and allowed to sediment for 30 min. Following repeated washing and centrifugation at 500× *g* for 5 min, the resulting sediment was examined microscopically. *Fasciola* spp. eggs were identified as large (130–150 × 60–90 µm), operculated, yellowish-brown eggs, whereas *D. dendriticum* eggs were identified as small (35–45 × 20–30 µm), thick-shelled, dark-brown, embryonated eggs [10,12,13]. Each preparation was independently evaluated by two parasitologists, and any discrepancies were resolved by a third examiner.

### 2.2. Postmortem Liver and Bile Duct Examination and Sampling Procedure

Within the scope of this study, the livers and bile ducts of cattle and sheep were systematically examined postmortem during the slaughter process to determine the presence of liver trematodes. Postmortem examination served as the primary reference method for parasitological diagnosis, as it allows for the direct detection of adult parasites and provides a reliable assessment of infection status. In the first stage of the examination, the external surface of the livers and the Glisson’s capsule were evaluated by inspection and palpation for color changes, capsular fibrosis, nodular formations, bile duct dilation, hepatomegaly, and other macroscopic pathological findings. Subsequently, longitudinal deep incisions were made along the common bile duct and major intrahepatic bile ducts to investigate the presence of adult trematodes within the lumen.

Following this, the liver parenchyma was sectioned into slices approximately 1–3 cm thick, encompassing the deep bile ducts and parenchymal migration tracts. The obtained tissue sections were manually pressed and washed in physiological saline (0.9% NaCl) to facilitate the recovery of small trematodes remaining within the deep bile ducts and tissue sections. All adult trematodes collected from the bile ducts and tissue washings were rewashed with sterile saline to remove tissue debris, and the presence or absence of adult parasites was recorded for each animal.

### 2.3. Morphological Identification and Preservation

The adult trematode specimens obtained were preserved in 70% ethanol at room temperature for morphological examination and taxonomic identification. Species-level diagnosis was performed using standard parasitological identification keys, taking into account the parasites’ general morphometric characteristics, body conformation, presence of the anterior conical projection and shoulder spines, arrangement of the testes, and organization of internal anatomical structures.

In accordance with these criteria, *Fasciola* specimens were identified at the genus level and recorded as *Fasciola* spp. based on their leaf-shaped body, distinct anterior conical projection, prominent shoulders, and approximate adult length of 2–3 cm. *D. dendriticum* was identified based on its small, flattened, lanceolate body, approximate length of 0.5–1 cm, and characteristic brownish, semi-transparent appearance. Its predominant localization in the smaller or distal bile ducts was considered a supportive finding rather than a morphological identification criterion [10,12,13,14].

### 2.4. Statistical Analysis

The categorical variables addressed in the study are presented as frequencies (n) and percentages (%). As the sample sizes were large and proportions were not near the extremes, the Z-test was considered appropriate. For paired binary data (comparison of fecal and postmortem examination results from the same animals), McNemar’s test with continuity correction was applied. For independent group comparisons (cattle vs. sheep, age groups, breed groups), the two-tailed Z-test for two proportions was used. Statistical significance was set at *p* < 0.05. For multiple comparisons in breed and age analyses (Table 3), the significance level was adjusted using Bonferroni correction (α’ = 0.05/8 = 0.00625) where applicable. Multivariable logistic regression analysis was performed to identify factors associated with *Fasciola* spp. and *D. dendriticum* infections. Age and breed were included as independent variables in the models for cattle and sheep separately. For cattle, age was categorized as 0–2 years and >2 years, and breed as Native Black and Holstein. For sheep, age was categorized as 0–2 years and >2 years, and breed as Akkaraman and Morkaraman. The reference categories were 0–2 years for age, Native Black for cattle breed, and Akkaraman for sheep breed. Adjusted odds ratios (OR) with 95% confidence intervals (CI) were calculated. Interaction terms (age × breed) were tested and removed from the final models when not statistically significant (*p* > 0.05). Model fit was assessed using the Hosmer-Lemeshow goodness-of-fit test. Data analysis was performed using SPSS (version 27) and Minitab (version 16).

During the preparation of this manuscript, the authors used DeepSeek (version v3, 2025-03) solely for language polishing, grammar correction, and improving sentence clarity.

## 3. Results

All adult *Fasciola* specimens were identified at the genus level and recorded as *Fasciola* spp.; species-level differentiation (e.g., between *F. hepatica* and *F. gigantica*) was not performed due to the lack of molecular confirmation. Therefore, all findings are reported as *Fasciola* spp. All animals in which *Fasciola* spp. eggs were detected by fecal examination also harbored adult *Fasciola* spp. at postmortem inspection. The prevalence of liver trematode infections in cattle and sheep was compared using fecal and postmortem examination methods. Based on postmortem examination, *Fasciola* spp. and *D. dendriticum* were detected in 26.2% (n = 98) and 7.2% (n = 27) of the 374 cattle examined, respectively. In sheep (n = 1837), postmortem inspection revealed *Fasciola* spp. infection in 14.8% (n = 271) of the animals, while *D. dendriticum* was identified in 5.9% (n = 108). Regarding fecal examination, *Fasciola* spp. and *D. dendriticum* were detected in 21.1% (n = 79) and 2.1% (n = 8) of cattle, respectively. In sheep, the fecal examination results indicated that 12.8% (n = 235) were positive for *Fasciola* spp. and 4.3% (n = 79) for *D. dendriticum* (Table 1).

The sensitivity of fecal sedimentation relative to postmortem examination was 80.6% (79/98) for *Fasciola* spp. and 29.6% (8/27) for *D. dendriticum* in cattle; and 86.7% (235/271) for *Fasciola* spp. and 73.1% (79/108) for *D. dendriticum* in sheep. The findings presented in Table 1 indicate that the total positivity infection values determined by postmortem examination was higher than that obtained by fecal examination in both animal species. In cattle, the total positivity infection values determined by postmortem examination (33.4%) was significantly higher than that obtained by fecal examination (23.3%). McNemar test revealed a statistically significant difference between the two methods for overall trematode detection (*p* < 0.001).

When individual parasite species were examined separately, postmortem examination detected significantly more infections than fecal sedimentation for both *Fasciola* spp. and *D. dendriticum* in both animal species (McNemar test, *p* < 0.001 for all comparisons).

The lower sensitivity of fecal sedimentation for *D. dendriticum*, particularly in cattle, may be partly explained by the smaller size of its eggs (approximately 35–45 × 20–30 µm) compared with those of *Fasciola* spp. (approximately 130–150 × 60–90 µm), which may increase the likelihood of egg loss during the decantation steps of the sedimentation procedure. Based on postmortem examination, *Fasciola* spp. prevalence and overall liver trematode positivity were significantly higher in cattle than in sheep (*p* < 0.001 for both comparisons). In contrast, the prevalence of *D. dendriticum* did not differ significantly between cattle and sheep (*p* = 0.274; z = 1.09) (Table 2).

In Table 2, *Fasciola* spp. infection was determined to be statistically significantly higher in cattle (26.2%) than in sheep (14.8%) (*p* = 0.001; z = 5.20). In contrast, no significant difference was found between cattle (7.2%) and sheep (5.9%) regarding *D. dendriticum* infection (*p* = 0.274; z = 1.09). The odds ratio analysis supported these findings. Cattle had approximately twice the odds of *Fasciola* infection compared to sheep (OR = 2.05, 95% CI: 1.58–2.66), indicating a clinically relevant difference in infection risk between the two host species. For *D. dendriticum*, the odds ratio was 1.24 (95% CI: 0.80–1.92), which was not statistically significant, as the confidence interval included 1.0. These findings indicate that the observed prevalence and odds of *Fasciola* spp. infection were higher in cattle than in sheep within the sampled slaughter population.

**Table 3:** Distribution of *Fasciola* spp. and *Dicrocoelium dendriticum* infections according to breed and age in cattle and sheep (univariable comparisons; adjusted odds ratios from the multivariable models are reported in the Results and Abstract).

Animal Species	Variable	Category	n (%)	*Fasciola* spp. n (%)	*p*-Value †	*Dicrocoelium* *dendriticum* n (%)	*p*-Value †
Cattle	Breed	Native Black	265 (70.9)	65 (24.5)	0.258	18 (6.8)	0.638
		Holstein	109 (29.1)	33 (30.3)		9 (8.3)	
	Age	0–2 years	192 (51.3)	11 (5.7)	<0.001	7 (3.6)	0.004
		>2 years	182 (48.7)	87 (47.8)		20 (11.0)	
Sheep	Breed	Akkaraman	938 (51.1)	186 (19.8)	<0.001	87 (9.3)	<0.001
		Morkaraman	899 (48.9)	85 (9.5)		21 (2.3)	
	Age	0–2 years	1281 (69.7)	203 (15.8)	0.043 *	61 (4.8)	0.002
		>2 years	556 (30.3)	68 (12.2)		47 (8.5)	

† The n (%) values and *p*-values shown in this table are from univariable two-tailed Z-tests for two proportions comparing categories within each variable (breed or age), calculated prior to Bonferroni correction; the Bonferroni-corrected significance threshold was α’ = 0.00625 (see Section 2.4). An asterisk (*) denotes a *p*-value below the nominal 0.05 threshold that did not remain significant after this correction. Adjusted odds ratios (OR) with 95% confidence intervals (CI) from the multivariable logistic regression models (age and breed entered simultaneously, so that each OR is adjusted for the other covariate) are reported in the Results and Abstract for the associations that remained statistically significant: cattle age (*Fasciola* spp. adjusted OR = 15.82, 95% CI: 8.12–30.84, *p* < 0.001; *D. dendriticum* adjusted OR = 3.29, 95% CI: 1.36–7.96, *p* = 0.008), sheep breed (*Fasciola* spp. adjusted OR = 0.42, 95% CI: 0.32–0.56, *p* < 0.001; *D. dendriticum* adjusted OR = 0.23, 95% CI: 0.14–0.38, *p* < 0.001, for Morkaraman versus Akkaraman), and sheep age for *D. dendriticum* only (adjusted OR = 1.84, 95% CI: 1.25–2.71, *p* = 0.002). Cattle breed and sheep age for *Fasciola* spp. were not statistically significant in the adjusted models. Interaction terms (age × breed) were tested and were not statistically significant (*p* > 0.05 for all models), indicating that the effect of age did not differ significantly between breeds. Hosmer-Lemeshow goodness-of-fit test *p* > 0.05 for all models. Reference categories: For age: 0–2 years; for breed in cattle: Native Black; for breed in sheep: Akkaraman.

The distribution of trematode infections concerning host breed and age is summarized in Table 3. Out of 374 examined cattle, 70.9% (n = 265) were Native Black and 29.1% (n = 109) were Holstein. The prevalence of *Fasciola* spp. was higher in Holsteins (30.3%) than in Native Black cattle (24.5%); however, this difference was not statistically significant (*p* = 0.258). Similarly, no significant variation was observed in *D. dendriticum* prevalence between Holstein (8.3%) and Native Black (6.8%) breeds (*p* = 0.638). In contrast, age was identified as a highly critical determinant for both parasitic infections in cattle. Cattle aged >2 years exhibited a remarkably higher prevalence of *Fasciola* spp. compared to the 0–2 years age group. A similar age-related trend was observed for *D. dendriticum*, where the prevalence significantly increased from 3.6% in younger animals to 11.0% in older ones (*p* = 0.004).

Among the 1837 sheep examined, 51.1% (n = 938) were Akkaraman and 48.9% (n = 899) were Morkaraman. Unlike the findings in cattle, host breed served as a significant risk factor for both trematode infections in sheep (*p* < 0.001). Akkaraman sheep displayed substantially higher infection values for both *Fasciola* spp. (19.8% vs. 9.5%) and *D. dendriticum* (9.3% vs. 2.3%) compared to Morkaraman sheep. Furthermore, age-associated variations in prevalence were statistically significant for both parasites in sheep. Interestingly, *Fasciola* spp. infection was numerically higher in younger sheep aged 0–2 years than in those aged >2 years (15.8% vs. 12.2%; *p* = 0.043); however, this difference did not reach statistical significance after Bonferroni correction for multiple comparisons (corrected α’ = 0.00625; see Section 2.4), and should therefore be interpreted as a non-significant trend rather than a true age effect on *Fasciola* spp. prevalence in sheep. Conversely, the prevalence of *D. dendriticum* followed the opposite pattern, rising significantly from 4.8% in the younger cohort to 8.5% in animals older than 2 years (*p* = 0.002).

Since the majority of the sample consisted of female animals, the sex distribution of infection could not be evaluated.

## 4. Discussion

Liver trematodes cause significant productivity losses in livestock production in regions where cattle and sheep farming are intensively practiced, zoonotic liver trematodes may also pose a potential risk to public health [15,16]. A comprehensive elucidation of the factors influencing the regional distribution of liver trematodes is of strategic importance for the development of veterinary medicine practices and public health policies within the “One Health” concept [17]. The presentation of reliable, up-to-date data from epidemiological research on liver trematodes provides a scientific basis for developing effective, sustainable control strategies. In this context, determining the prevalence of liver trematodes in cattle and sheep examined during slaughter is important for understanding regional parasitic infection dynamics and developing disease control strategies.

The evidence-based findings from this context strengthen the scientific infrastructure of regional parasite control programs, thereby directly contributing to sustainable policies that improve animal welfare and minimize public health risks [18].

The comparison of diagnostic methods is a central theme of the present study, as the choice of screening tool directly affects reported prevalence values and subsequent control decisions. Fecal sedimentation is widely used under field conditions because of its low cost and simplicity; however, its sensitivity may be reduced during prepatent infections, in animals with low parasite burdens, and when egg shedding is limited or intermittent [9]. Postmortem examination, in contrast, allows for the direct recovery and morphological identification of adult trematodes and was therefore used as the reference method in the present study. However, this approach cannot be used for large-scale screening of live animals.

The sensitivity of methods used to diagnose liver trematodes is important for the reliability of true-positive values in the field. Scientific research has demonstrated that postmortem examination findings are more sensitive than microscopic examination [19]. In the present study, postmortem examination of the liver, gallbladder, and bile ducts was used as the reference method for evaluating infection status in slaughtered animals. However, these findings should be interpreted as observed postmortem detection values rather than absolute true-positive results. Various studies worldwide and in Türkiye have reported data on the prevalence of liver trematode infections. Globally, the prevalence of *F. hepatica* in sheep varies widely, from as low as 0.7% in Egypt and Iraq to as high as 58.5% in Pakistan and 45.7% in Ethiopia [20,21]. Intermediate prevalence values have been reported from China (14.98%), Mexico (19.4–30.6%), and Bangladesh (27%) [22].

Epidemiological studies conducted in sheep across different regions of Türkiye show that infection values for liver trematodes range from 3.99% to 72.6% for *F. hepatica* and from 1.15% to 68.6% for *D. dendriticum* [15,23,24,25,26,27,28].

In a meta-analysis, 371 articles were evaluated, and the average infection values of fasciolosis were estimated at 17% in cattle, 13% in sheep, and 5% in humans [22]. In this study, our team observed adult *Fasciola* spp. were detected in 14.8% (271/1837) of sheep by postmortem examination. This infection value is similar to the average value detected in sheep by Lan et al. [22].

Epidemiological studies on cattle in different countries show that the prevalence values of liver trematodes vary considerably by region. In a meta-analysis, the global mean prevalence of fasciolosis in cattle was reported as 17%. However, infection values reported across studies range from 12.02% to 96.67%, depending on continent [22]. In the present study, *Fasciola* spp. were detected postmortem in 26.2% of cattle, which was higher than both the reported global mean and the 14.8% value observed in sheep in this study. The observed prevalence of *Fasciola* spp. was significantly higher in cattle than in sheep among the animals examined during slaughter. However, environmental and management-related determinants were not directly evaluated and therefore cannot be established from the present data.

In regions where extensive, pasture-based animal husbandry predominates, the risk of bovine fasciolosis is high [29]. Despite regional differences, the 26.2% prevalence of *Fasciola* spp. detected in cattle in the present study appears epidemiologically consistent with the 23.5% infection value reported by Şahin et al. [30] in the Ağrı region. This similarity may reflect comparable ecological or husbandry conditions; however, such factors were not directly assessed in the present study. Therefore, the findings indicate substantial exposure to *Fasciola* spp. among slaughtered cattle in Siirt but are insufficient on their own to establish that the entire region is endemic.

When examining the literature on ruminants, it is evident that existing data on the epidemiology of dicrocoeliosis are significantly more limited than findings on fasciolosis. Investigating the presence of *D. dendriticum* in sheep and cattle in this study is important for a holistic evaluation of liver trematodes. In this study, the prevalence of *D. dendriticum* in sheep and cattle in the Siirt region was determined as 5.9% and 7.2%, respectively. These infection values show marked differences when compared with data reported from other studies in Türkiye. Studies on the epidemiology of *D. dendriticum* in sheep and cattle in Türkiye indicate high infection values in endemic regions such as Eastern Anatolia and Marmara. In this context, a review of the existing literature reports prevalence values ranging from 41.0% in the Kars region [31] to 64.0–74.0% in the Southern Marmara region [32] in sheep. The high infection values are attributed to climatic dynamics, pasture structure, and the density of intermediate host populations in these regions. The infection prevalence detected in the sheep (5.9%) and cattle (7.2%) populations in the Siirt region of our study is markedly lower than the infection values reported in these hyperendemic regions. In contrast, infection values reported from Malatya province (3.85% in sheep, 4.67% in cattle) [25] were closer to our findings. However, because the present study was based on animals examined during slaughter, these findings are insufficient to classify the overall risk level of Siirt Province as medium or low. Comparisons among regions should also be interpreted cautiously because of differences in sampling designs, animal populations, study periods, and diagnostic methods.

Another specific finding in our study is that the frequency of *D. dendriticum* in cattle (7.2%) was higher than in sheep (5.9%). In contrast, the literature generally reports higher infection values of parasitic infection in sheep than in cattle. Previous studies have frequently reported higher prevalence values in sheep than in cattle, potentially reflecting differences in grazing behaviour and contact with intermediate hosts [33,34]. However, it is anticipated that specific geographical and ecological factors unique to the Siirt region may have given rise to this difference. Although we did not collect environmental data, one hypothesis worth investigating is that cattle in Siirt may be grazed more frequently in low-lying, humid areas that support higher populations of intermediate hosts (*Lymnaea* snails for *Fasciola* and formicine ants for *Dicrocoelium*). A targeted malacological and myrmecological survey would be necessary to test this hypothesis. Other studies, such as one conducted in Malatya, where the prevalence of dicrocoeliosis in cattle was found to be similar to or higher than in sheep, also support the existence of such regional epidemiological dynamics.

Studies on this subject report that puddles on high-altitude and sloped terrain optimize snail population dynamics in the family *Lymnaeidae*, which play a significant role in the parasite life cycle [35,36].

Cattle had a significantly higher observed prevalence of trematode infection than sheep among the animals examined during slaughter in the Siirt region. The observed difference between cattle and sheep may be associated with ecological and management-related factors; however, these factors were not directly evaluated in the present study [36]. The substantially higher prevalence and odds of fasciolosis observed in cattle (approximately twice that of sheep) has important practical implications. Control programs in the region should prioritize cattle herds for targeted anthelmintic treatment and pasture management, as they exhibited the highest observed prevalence and odds of infection among the host species sampled in this study.

The strong association between age and fasciolosis prevalence in cattle supports the ‘cumulative exposure’ theory [37]. As cattle age, they spend more time on pasture, increasing their cumulative risk of exposure to infective metacercaria. The failure to develop a fully protective immune response against *Fasciola* spp. [38,39] allows repeated infections to accumulate, leading to higher parasite burdens and prevalence in older animals. This age-related pattern has important implications for control: younger cattle (under 2 years) may be targeted for chemoprophylaxis before turn-out, while older animals may require treatment based on diagnostic testing.

The low sensitivity of fecal sedimentation for *D. dendriticum*, particularly in cattle (29.6%), represents a major diagnostic gap. The smaller size of *Dicrocoelium* eggs (approximately 40 × 25 μm) compared with *Fasciola* eggs (150 × 90 μm) may contribute to their loss during the decantation steps of the sedimentation procedure; however, this mechanism was not directly evaluated in the present study and should not be considered the sole explanation [10,12]. Similar underestimation of trematode prevalence by coprological methods has been reported in other settings; Mathewos et al. [19] found that fecal examination underestimated bovine fasciolosis by approximately 35% compared to necropsy in Ethiopia. The global sensitivity of fecal sedimentation for *Fasciola* spp. has been estimated at 70–85% [22], which aligns with our finding of 80.6% in cattle. However, to our knowledge, quantitative sensitivity data for *Dicrocoelium* are scarce; our finding of 29.6% highlights a previously underappreciated diagnostic challenge. From a practical perspective, field surveys relying solely on fecal sedimentation may substantially underestimate *Dicrocoelium* prevalence, particularly in cattle. Where validated and available, molecular or serological methods may be considered as complementary diagnostic approaches.

In contrast, *Fasciola* spp. prevalence in sheep was numerically higher in the 0–2 years age group (15.8%) compared to animals aged 2 years and over (12.2%); however, as noted in the Results, this difference did not remain statistically significant after Bonferroni correction and was not confirmed in the multivariable model. If replicated in future studies with adequate power, one possible explanation could be that a specific protective immune response against the parasite in ruminants only fully develops at advanced ages [38,39].

Another factor contributing to the low prevalence infection values observed in adult sheep is the “pathological masking” mechanism. Severe fibrosis of the liver parenchyma in older animals and intense calcification of the bile ducts can mechanically encapsulate adult trematodes, terminating parasite viability and suppressing egg production. This fibrotic barrier may lead to underestimation of infection levels, especially in older age groups, during both coproscopic and macroscopic examinations [40].

A notable characteristic of our study sample was the heavy predominance of female animals, a distribution that directly mirrors the breeding-oriented farming models of the region. In local livestock management, female animals are kept for multiple productive cycles, leading to prolonged and cumulative pasture exposure across successive grazing seasons. In contrast, male animals are typically raised in enclosed feedlots and slaughtered at a younger age, minimizing their contact with infective metacercaria. Furthermore, the physiological stress of pregnancy and lactation may suppress maternal immunity, which could plausibly contribute to greater susceptibility to parasitic establishment in females, although this hypothesis was not directly tested in the present study. While this female-skewed distribution accurately reflects the actual field dynamics, the underrepresentation of fattening males remains a methodological limitation. Nevertheless, this extensive screening across different host species offers a highly reliable epidemiological baseline for regional hepatobiliary infections [41,42].

Although sporadic human fasciolosis cases have been reported in Türkiye [43], the detection of *Fasciola* spp. in 26.2% of cattle and 14.8% of sheep slaughtered in Siirt does not directly demonstrate environmental contamination with metacercariae or the magnitude of human infection risk in the province. Moreover, the animals’ farm or district of origin was not systematically recorded. Humans acquire fasciolosis primarily through the consumption of raw aquatic plants, such as watercress (*Nasturtium officinale*), contaminated with metacercariae. Therefore, the present findings support general awareness of zoonotic fasciolosis and the risks associated with consuming raw aquatic plants from potentially contaminated areas. However, local dietary practices, environmental contamination, and human fasciolosis cases were not investigated; consequently, Siirt-specific exposure patterns and targeted public health interventions cannot be inferred from the present study.

In our study evaluating sheep breeds, the Akkaraman breed had significantly higher odds of infection with both liver trematode taxa than the Morkaraman breed. However, this association does not demonstrate that Akkaraman sheep are genetically more susceptible to infection. Breed may be associated with unmeasured differences in geographical origin, grazing practices, environmental exposure, or herd management. Because these factors were not evaluated, the biological or environmental mechanisms underlying the observed breed association could not be determined [44,45].

This study has several limitations. First, the absence of histopathological examination prevented direct assessment of the ‘pathological masking’ hypothesis proposed to explain the lower *Fasciola* prevalence in older sheep. Second, we did not perform quantitative egg counts (e.g., eggs per gram of feces), which would have allowed us to correlate egg shedding intensity with adult worm burden. Third, environmental data (e.g., snail and ant populations, temperature, humidity) were not collected, limiting our ability to draw firm conclusions about ecological risk factors. Fourth, the sex distribution of infection could not be evaluated because female animals predominated in the sample. Finally, sampling conducted during slaughter may not fully represent the live animal population in the region, as animals selected for slaughter may differ from the general herd in terms of age, health status, or management conditions. Additionally, information on the animals’ district or farm of origin was not systematically recorded during sample collection. Consequently, we were unable to assess the spatial distribution of infections within Siirt province. The heavy predominance of female animals in our sample (cattle: 92.5%; sheep: 94.1%) reflects the breeding-oriented farming models of the region, where female animals are kept for multiple productive cycles, leading to prolonged pasture exposure. In contrast, male animals are typically raised in feedlots and slaughtered at younger ages, limiting their contact with infective metacercariae. While this female-skewed distribution accurately reflects local farming practices, it prevented a statistical evaluation of sex-related differences in infection prevalence, representing an additional limitation of this study. Similar sex imbalances have been reported in other slaughterhouse-based studies [41,42]. Future studies should incorporate geographic information to identify high-risk areas and facilitate more targeted control interventions.

## 5. Conclusions

In conclusion, the high detected levels of liver trematodes, particularly *Fasciola* spp. in cattle, among animals slaughtered in Siirt indicates that these infections remain an important veterinary concern in the study population. However, the study design based on animals being examined during slaughter does not allow the findings to be generalized to the entire livestock population of the province. Effective control should include regular veterinary surveillance, evidence-based anthelmintic treatment, and management practices aimed at reducing exposure to intermediate hosts. For fasciolosis, grazing in wet habitats that favor lymnaeid snail populations should be managed carefully, whereas control of dicrocoeliosis should consider the involvement of terrestrial snails and ants in the life cycle of *D. dendriticum*. Postmortem examination detected significantly more infections than fecal sedimentation for both *Fasciola* spp. and *D. dendriticum* in cattle and sheep, indicating that reliance solely on fecal sedimentation may lead to an underestimation of prevalence. With regard to zoonotic fasciolosis, increasing public awareness about avoiding the consumption of raw aquatic plants collected from potentially contaminated areas and improving healthcare professionals’ awareness of possible sporadic human cases may contribute to reducing public health risks.

## Figures and Tables

**Table 1:** Comparison of fecal examination and postmortem inspection for detection of *Fasciola* spp. and *D. dendriticum* in cattle and sheep.

Animal Species	Examination Method	*Fasciola* spp. n (%) [95% CI]	*D. dendriticum* n (%) [95% CI]
Cattle (n = 374)	Fecal examination	79 (21.1) [17.1–25.6]	8 (2.1) [0.9–4.2]
	Postmortem inspection	98 (26.2) [21.8–31.1]	27 (7.2) [4.8–10.3]
	*p*-value (McNemar) †	<0.001	<0.001
Sheep (n = 1837)	Fecal examination	235 (12.8) [11.3–14.4]	79 (4.3) [3.4–5.3]
	Postmortem inspection	271 (14.8) [13.2–16.5]	108 (5.9) [4.9–7.1]
	*p*-value (McNemar) †	<0.001	<0.001

† McNemar test with continuity correction for paired binary data (same animals examined by both methods). Postmortem inspection was considered the reference method. All comparisons showed statistically significant differences between fecal sedimentation and postmortem examination (*p* < 0.001). 95% CIs were calculated using the exact binomial method.

**Table 2:** Comparison of liver trematode prevalence between cattle and sheep based on postmortem examination.

Parasite Species	Cattle (n = 374) n (%) [95% CI]	Sheep (n = 1837) n (%) [95% CI]	*p*-Value (z-Value) †	Odds Ratio (95% CI)
*Fasciola* spp.	98 (26.2) [21.8–31.1]	271 (14.8) [13.1–16.4]	0.001 (5.20)	2.05 (1.58–2.66)
*D. dendriticum*	27 (7.2) [4.8–10.3]	108 (5.9) [4.9–7.1]	0.274 (1.09)	1.24 (0.80–1.92)

† Two-tailed Z-test for two proportions comparing cattle vs. sheep. Postmortem examination was used as the reference method.

## Data Availability

The original contributions presented in this study are included in the article. Further inquiries can be directed to the corresponding author.
